# Lessons Learned From Presumptive Condition Lists in Veteran Compensation Systems

**DOI:** 10.3389/fpubh.2022.739746

**Published:** 2022-05-10

**Authors:** Amy L. Hall, Paul A. Demers, Linda VanTil, Mary Beth MacLean, Maria E. Dalton, Trish Batchelor, Lesley Rushton, Tim R. Driscoll

**Affiliations:** ^1^Research Directorate, Veterans Affairs Canada, Charlottetown, Prince Edward Island, PE, Canada; ^2^Occupational Cancer Research Centre, Cancer Care Ontario, Toronto, ON, Canada; ^3^Faculty of Health Sciences School of Rehabilitation Therapy, Queens University, Kingston, ON, Canada; ^4^Cumming School of Medicine, University of Calgary, Calgary, AB, Canada; ^5^Department of Veterans' Affairs, Canberra, ACT, Australia; ^6^School of Public Health, Faculty of Medicine, Imperial College London, London, United Kingdom; ^7^Sydney School of Public Health, Sydney Medical School, The University of Sydney, Sydney, NSW, Australia

**Keywords:** veteran health, veteran affairs, veteran benefits, scientific review, occupational compensation, veteran health administration

## Abstract

Presumptive condition lists formally accept connections between military factors and veteran health conditions. An environmental scan of such lists and their evidentiary basis was conducted across four veterans' administrations to inform other administrations considering the development of such lists. Information on included conditions, qualifying military factors, and scientific processes was obtained through targeted internet searches and correspondence with veterans' administrations. The content of presumptive condition lists across jurisdictions varied by conditions included, as well as military eligibility requirements (e.g., service in particular conflict, context, or time period). Scientific review processes to develop lists also varied across jurisdictions. Findings indicate that evidence and experience may be leveraged across compensation systems (veteran and civilian). Ongoing research to understand links between military exposures and veteran health is recommended.

## Introduction

As our understanding of occupational hazards and their health impacts evolves, so does recognition of the need to compensate individuals affected. However, determinations of which conditions are compensable, and under what circumstances, are often not straightforward.

The complexities of establishing work-related causality are well demonstrated in military veteran compensation systems. When adjudicating a veteran's benefit application, the decision-maker must review and weigh different types of evidence to determine the relatedness of a claimed condition to military service. This process requires the gathering, analysis and weighing of past military service, exposure, and medical records, current scientific and medical evidence on occupational exposures and diseases, and relevant personal information.

Determinations of entitlement in this context can be challenging, since an individual's military service may include a range of occupational circumstances and hazards with subsequent impacts on various health conditions (e.g., mental, musculoskeletal, and other chronic outcomes) (1–4). While some hazards are similar to those found in civilian workplaces (e.g., diesel engine exhaust, temperature, and noise extremes), others are unique to military service. Examples of the latter include exposures during overseas deployments (e.g., burn pit smoke and infectious agents), combat (e.g., chemical warfare agents and depleted uranium), and basic training (e.g., extreme physical loads) (1, 2). The challenge of isolating the occurrence and effects of military exposures is further complicated by time lags between release from active service and application for veteran benefits, variable quality and accessibility of military records, and long latency periods for many chronic health conditions (2).

To streamline the benefits application process, veteran compensation systems in some countries, such as the United States of America (5), the United Kingdom (6, 7), Australia (8), and New Zealand (9), have developed presumptive condition (sometimes referred to as “automated” or “streamlined”) lists appearing either in schedules to legislation or supporting regulations. If a veteran diagnosed with a listed condition has been exposed to the corresponding military activity, the condition is presumed to be related to military service.

By reducing burden of proof requirements, presumptive condition schedules offer potential advantages of streamlined disability benefit processing and expedited decision making. However, determinations of which health conditions are presumptively compensated through veteran benefit systems, and under what circumstances, is a complex process informed by legal principles and, ideally, by scientific principles of causality.

This article intends to provide an overview of presumptive condition lists and scientific procedures underpinning their development, across four veteran compensation systems. Key scientific procedures and principles that support the development of veteran-focused presumptive condition lists are discussed, as well as opportunities for knowledge exchange across veteran and civilian systems. This information on scientific practices and principles underlying presumptive condition lists is meant to serve as a starting point for administrations tasked with establishing or expanding such lists within their own systems.

## Methods

Environmental scans are used in governmental and business contexts to gather and interpret information to inform strategic decision making and to direct organizational action (10). The current environmental scan was conducted by Veterans Affairs Canada in the summer of 2020 to collect information on presumptive condition lists (conditions included and military exposure criteria for presumption to apply) and the scientific procedures used to inform them. Four veterans' administrations, representing English speaking countries with a range of population sizes and geographic locations, were included: The United States of America (US), The United Kingdom (UK), Australia (AU), and New Zealand (NZ).

The scope of the scan was delineated by the following terms: “veteran presumptive conditions,” “veteran compensation,” “veteran health,” and “occupational conditions veterans.” The scan sought to identify health conditions included, prerequisites or criteria for entitlement, and scientific processes used for their selection. The scan did not include a comparison of the type of compensation paid (e.g., functional impairment), the types of benefits payable (e.g., disability award), or other procedural issues (e.g., treatment approaches, acceptance rates).

Enabling statutes and downstream policy instruments were searched and retrieved through official websites of the organization with responsibility for (or oversight of) veterans' benefits (The United States Department of Veterans Affairs, The United Kingdom's Ministry of Defense, Australia's Department of Veterans' Affairs, and Veterans' Affairs New Zealand), and other websites and online portals with details on relevant legislation and policy. Correspondence with research-affiliated individuals working within the relevant administration was also sought, for supplemental information.

All presumptive compensation statutes in the US, UK, AU, and NZ (as well as the regulations and policies made pursuant to the legislation) were examined to identify any relevant information regarding coverage (presumptive or otherwise) for health-related injuries, conditions and/or impairment. Where a piece of legislation, regulation or policy appeared to be relevant to the scan, information on conditions included, eligibility criteria, and scientific review (undertaken for the determination of conditions and eligibility criteria) was extracted and recorded.

This work was performed within the Research Directorate of Veterans Affairs Canada, Charlottetown, Prince Edward Island, Canada. Ethics approval was not required for this environmental scan of public data sources.

## Results

Conditions included within presumptive condition lists (or their equivalent), and their associated exposure criteria, across the US, UK, AU, and NZ are summarized in Tables 1–4, respectively. Cancers are the most widely presumptively covered types of conditions across countries, particularly in relation to Vietnam War service. Mental health conditions are also included in most presumptive lists, with the exception of the UK. Skin conditions are widely covered, with AU addressing a wider range of such conditions. The US and AU outline the greatest number of conditions/condition groupings, with differing exposure criteria and limits (8, 11).

**Table 1:** Included conditions and exposure criteria for veterans, The United States of America (5, 11)^a^.

**Condition**	**Exposure criteria**
Anemia, primary; Arteriosclerosis; Arthritis; Atrophy, Progressive muscular; Brain hemorrhage; Brain thrombosis; Bronchiectasis; Calculi of the kidney, bladder, or gallbladder. Cardiovascular-renal disease including hypertension; Cirrhosis of the liver; Coccidioidomycosis; Diabetes mellitus; Encephalitis lethargica residuals; Endocarditis; Endocrinopathies; Epilepsies; Hansen's disease; Hodgkin's disease; Leukemia; Lupus erythematosus, systemic; Myasthenia gravis; Myelitis; Myocarditis; Nephritis; Other organic nervous system diseases; Paget's disease; Osteomalacia; Palsy, bulbar; Paralysis agitans; Psychoses; Purpura idiopathic, hemorrhagic; Raynaud's disease; Sarcoidosis; Scleroderma; Sclerosis, amyotrophic lateral or multiple; Syringomyelia; Thromboangiitis obliterans; Tuberculosis, active; Tumors, malignant, or of brain, spinal cord or peripheral nerves; Ulcers, peptic (gastric or duodenal).	Condition becomes manifest within 1 year (within 3 years for Hansen's disease and tuberculosis; within 7 years for multiple sclerosis) from the date of separation from service.
Amebiasis; Blackwater fever; Cholera; Dracontiasis; Dysentery; Filariasis; Leishmaniasis; Loiasis; Malaria; Onchocerciasis; Oroya fever; Pinta; Plague; Schistosomiasis; Yaws; Yellow fever; Resultant disorders or diseases originating from therapy administered in connection with such diseases or as a preventative.	Tropical service.
Avitaminosis; Beriberi (including beriberi heart disease); Chronic dysentery; Helminthiasis; Malnutrition (including associated optic atrophy); Pellagra; Other nutritional deficiencies; Irritable bowel syndrome^a^; Peptic ulcer disease; Peripheral neuropathy except where directly related to infectious cause; Cirrhosis of the liver (September 28, 2009 or after); Osteoporosis.	Former prisoner of war, imprisoned for at least 30 days.
Any of the anxiety states; Psychosis; Dysthymic disorder; Stroke and its complications; Cirrhosis of the liver; Atherosclerotic heart disease or hypertensive vascular disease and their complications; Organic residual of frostbite or trench foot; Post-traumatic osteoarthritis; Osteoporosis (specified circumstances).	Former prisoner of war, imprisoned for any length of time.
AL amyloidosis; Chloracne or other acneform; Type 2 diabetes; Hodgkin's disease; Ischemic heart disease; All chronic B-cell leukemias; Multiple myeloma; Non-Hodgkin's lymphoma; Parkinson's disease; Early-onset peripheral neuropathy; Porphyria cutanea tarda; Prostate cancer; Respiratory cancers (cancer of the lung, bronchus, larynx, or trachea); Soft-tissue sarcoma (other than osteosarcoma, chondrosarcoma, Kaposi's sarcoma, or mesothelioma).	Exposure to an herbicide agent during active military, naval, or air service; including service in the Vietnam War in the Republic of Vietnam between January 9, 1962, and May 7, 1975.
Multiple Myeloma, All forms of leukemia (other than chronic lymphocytic leukemia); lymphomas (except Hodgkin's disease); Primary liver cancer (except if cirrhosis or hepatitis B is indicated); Bronchiolo-alveolar carcinoma; Cancer of the bile ducts, brain, breast, bone, colon, lung, gall bladder, esophagus, ovaries, pancreas, pharynx, salivary gland, small intestine, stomach, thyroid, or urinary tract (including kidneys, renal pelves, ureters, urinary bladder, and urethra).	Veterans who participated in a “radiation-risk” activity: Onsite participation in a test involving atmospheric detonation of a nuclear device; Occupation of Hiroshima or Nagasaki, Japan, between August 6, 1945, and July 1, 1946; Internment as prisoner of war in Japan (or service in Japan following internment) in WW2; Service before Feb 1, 1992 at specified US gaseous diffusion plants; Service before January 1, 1974 on Amchitka Island, Alaska; Qualification as “Special Exposure Cohort” member.
Cancer of the kidney, liver, or bladder; Non-Hodgkin's lymphoma; Adult leukemia; Multiple Myeloma; Parkinson's Disease; Aplastic anemia and other myelodysplastic syndromes.	Exposure to contaminants in the water supply at Camp Lejeune during service.
	Gulf War Veterans who:
Medically unexplained chronic multi-symptom illnesses that exist for 6 months or more, such as Chronic fatigue syndrome, Fibromyalgia, Irritable bowel syndrome; Any diagnosed or undiagnosed illness that warrants a presumption of service connection, as determined by the Secretary of Veterans Affairs	Served in the Southwest Asia Theater of Operations and have a condition that is at least 10% disabling by December 31, 2026
Brucellosis; Campylobacter jejuni; Coxiella burnetii (Q fever); Non-typhoid Salmonella; Shigella; West Nile virus; Malaria (or when accepted treatises indicate the incubation period began during a qualifying period of service); mycobacterium tuberculosis; visceral leishmaniasis	Served in the Southwest Asia Theater of Operations or in Afghanistan on or after September 19, 2001 and manifest one of the following infectious diseases to a degree of 10% or more within 1 year of separation (note: mycobacterium tuberculosis and visceral leishmaniasis covered at any time after separation)
Asthma; rhinitis; sinusitis, to include rhinosinusitis	Served any length of time in the Southwest Theater of Operations during the Persian Gulf War, or any length of time in Afghanistan, Syria, Djibouti or Uzbekistan on or after September 19, 2001, and manifests condition to any degree within 10 years of separation from military service

a *Abbreviated list and criteria; see references for full details on conditions and exposure criteria*.

**Table 2:** Included conditions and exposure criteria for veterans, The United Kingdom (6, 7).

**Condition**	**Exposure criteria**
Mesothelioma	Royal Navy service of any duration on seagoing ships between 1939 and 1973
Leukaemias (other than chronic lymphatic leukemia)	Participation at the tests or experimental programmes without case-specific dose determination, when presenting clinically within 25 years of presence at the tests or weapons experiments

**Table 3:** Included conditions and exposure criteria for Veterans, Australia (8).

	**Exposure criteria^**a**^**
**Condition—Streamlined**	
Non-melanotic malignant neoplasm of the skin; Malignant melanoma of the skin or eye; Acquired cataract; Benign neoplasm of the eye and adnexa; Malignant neoplasm of the eye; Non-melanotic malignant neoplasm of the skin; Pinguecula; Pterygium; Seborrheic keratosis; Solar keratosis	Sunlight or ultraviolet light exposure, various cumulative hours and geographic areas
Achilles tendonitis or bursitis; Chondromalacia patella; Femoroacetabular impingement syndrome; Iliotibial band syndrome; Patellar tendinopathy; Plantar fasciitis; Shin splints; Trochanteric bursitis and gluteal tendinopathy; Patellar tendinopathy	Weight bearing exercise involving repeated activity, various intensities
Acute articular cartilage tear; Acute meniscal tear of the knee; Labral tear; Sprain and strain; Dislocation of a joint; Cut, stab, abrasion and laceration; Fracture; Joint instability; External bruise; Internal derangement of the knee	Significant physical force or physical trauma applied to or through affected area
Physical injury due to munitions discharge	Munitions discharge
Sensory neural hearing loss; Tinnitus	Sound exposure of at least 140 dB(C)
External burn	Heat source, extreme cold, corrosive chemicals, radiation
Tinea	Exposed to the tinea dermatophyte; skin maceration; other
**Condition—straight-through**	
Intervertebral disc prolapse; Lumbar spondylosis; Osteoarthritis (lower limb); Thoracic spondylosis	Lifting loads, various cumulative load-factors
Rotator cuff syndrome	Repetitive or sustained activities of the affected shoulder
Post-Traumatic Stress Disorder (PTSD); Anxiety disorder; Adjustment disorder	Hostile or life threatening environment for at least 4 weeks, various onset timeframes

a *Abbreviated; see full summary of factors required to connect conditions with military service via (8)*.

**Table 4:** Included conditions and exposure criteria for veterans, New Zealand (9).

**Condition**	**Exposure criteria**
AL-type primary amyloidosis; Chloracne; Type 2 diabetes; Ischaemic heart disease; Hodgkin's disease; Hypertension; Non-Hodgkin's lymphoma; Chronic lymphocytic leukemia (including hairy-cell leukemia and other chronic B-cell leukaemias); Multiple Myeloma; Acute and subacute peripheral neuropathy; Parkinson's disease; Porphyria cutanea tarda; Prostate cancer; Respiratory cancers of the lung, bronchus, larynx, trachea; Soft-tissue sarcoma; Stroke	Served in Vietnam between May 29 1964 and March 1975
All forms of leukemia (except chronic lymphocytic leukemia); Lymphomas (other than Hodgkin's lymphomas); Multiple Myeloma; Primary liver cancer (except if cirrhosis or hepatitis B is indicated); Bronchioloalveolar carcinoma; Cancer of the bile ducts, brain, breast, bone, colon, lung, gall bladder, esophagus, ovary, pancreas, pharynx, salivary gland, small intestine, stomach, thyroid, or urinary tract (renal, ureter, urinary bladder, or urethra)	Exposed to nuclear radiation^a^
Chronic fatigue syndrome; Fibromyalgia; Irritable bowel syndrome	Served in the Gulf Conflict between December 20 1990 and April 13 1991
Any of the anxiety states; Beriberi; Chronic dysentery; Cirrhosis of the liver; Dysthymia; Heart disease or hypertensive vascular disease and their complications; Helminthiasis (intestinal vermiform parasites); Hypovitaminosis; Irritable bowel syndrome; Malnutrition (including optic atrophy); Organic residual of frostbite or trench foot; Pellagra and/or other nutritional deficiencies; Peptic ulcer disease; Peripheral neuropathy; Post-traumatic osteoarthritis; Psychosis; Stroke and residuals of stroke.	Prisoner of war, for any length of time, during the Second World War

a *Service as part of the British Occupation Force of Japan between 1946 and 1952 (J Force), or on HMNZS Pukaki from May 15, 1957 to November 8, 1957, HMNZS Pukaki from April 28, 1958 to September 23, 1958, HMNZS Otago on July 22, 1973, HMNZS Canterbury on July 28, 1973, HMNZS Rotoiti as part of Operation Grapple from May 15, 1957 to November 8, 1957*.

In terms of exposure criteria for presumptions to apply (also summarized in Tables 1–4), the US outlines circumstances based on factors such as time since release from service, locations, conflicts, and time periods served. NZ identifies conflicts, while The UK's presumptions relate to service contexts (i.e., nuclear testing and sea-faring service). In addition to some specified military conflicts/situations (i.e., being a former prisoner of war), AU outlines other types of exposure criteria by condition, including both service-related and non-service related exposures. Service- related exposures (summarized in this article) are associated with exposures that may be encountered during service, while non-service-related exposures are those that may be encountered during civilian life outside of service.

Scientific procedures used to develop presumptive condition lists are described in the following sections.

### The United States of America

The US Department of Veterans Affairs ensures that a review of scientific and medical evidence is completed prior to the inclusion of any condition on their list of presumptions. This review includes reports from the National Academies of Sciences, Engineering, and Medicine (12), as well as analyses/reviews conducted by scientific bodies within the Department.

The process to develop a presumption begins with a request by Post Deployment Health Services for a report from the National Academies of Sciences, Engineering, and Medicine. The National Academy secretariat convenes nationally recognized experts who perform a consensus review of the literature on links between exposures to potentially hazardous agents and health outcomes. This format has been used for a range of exposures and conditions, for example when reviewing human health evidence on the effects of exposure to Agent Orange (13) and antimalarial drugs (14). National Academies reports are subsequently reviewed by technical working groups within the US Department of Veterans Affairs. Thus, scientific evidence is reviewed externally and then internally to develop recommendations for Veterans Affairs.

### The United Kingdom

The UK War Pension Scheme (15) makes awards for disorders causally linked to service prior to April 6, 2005, when the Armed Forces Compensation Scheme (16) was introduced. There is no concept of prescription or presumption under the Armed Forces Compensation Scheme. However, under the War Pension Scheme there are two situations where presumption rather than case-by-case assessment may occur:

1) Entitlement for leukaemias (other than chronic lymphatic leukemia) is accepted without case-specific dose determination, when the affected person presents clinically within 25 years of presence at specified UK Atmospheric nuclear test and weapons experiments (7).
2) Entitlement for mesothelioma is based on a presumption of a service link where there is Royal Navy service of any duration on seagoing ships between 1939 and 1973 (6).

These presumptions are outlined in reports by the “Independent Medical Expert Group” (IMEG), tasked by the Minister for Defense Personnel and Veterans to investigate medical and scientific topics related to the Armed Forces Compensation Scheme. The IMEG is a non-departmental public body comprising independent consultants drawn from a number of medical specialties, which investigates the issues on which advice is requested, provides evidence-based conclusions and recommendations based on peer-reviewed scientific and medical literature (17). It consults other experts and invites interested parties to submit relevant research on an as-needed basis (18).

### Australia

Since 2007, Australia's Department of Veterans' Affairs has been developing systems to streamline and automate disability benefit processing, with 43 “decision ready” conditions processed through multiple streams to date. An important basis for these conditions are Statement of Principles (SOPs), legislative instruments with statements concerning which exposure factors must exist to establish a causal connection between military service and a particular health condition (19). Burdens of proof vary, for example combat service is associated with a lower burden of proof compared to non-combat service.

SOPs are developed by the AU Repatriation Medical Authority [an independent statutory authority responsible to the Minister for Veterans' Affairs (20)] through a global review of military and civilian evidence. If sufficient evidence is found to support a link between an exposure factor and condition, then this factor will be included in the SOP. While not presumptive, SOPs inform the development of decision-ready condition lists, with their listed exposure criteria used in application of the presumption in some instances. Lumbar spondylosis provides one example of a quantifiable SOP where a lifting factor is applied, with required periods of service ranging from 28 days for Special Forces members up to 1,360 days for Officers in the Royal Australian Air Force.

### New Zealand

Lists of conclusively presumed conditions linked to operational service in NZ were introduced as policy to direct decision-making in 2007, adapted from the US list of presumptive conditions. When the NZ Law Commission conducted an independent review of the War Pensions Act 1954 in 2010, it recommended the adoption of AU's Statements of Principles but did not recommend removing the lists of conclusively presumed conditions that had been adopted from the US. The current list of presumptive conditions (9) was accepted in 2014.

Veterans' Affairs NZ adopted AU's SOPs in December 2014 and established a Veterans' Health Advisory Panel to adopt or amend presumptive decision-making. As a result, NZ relies heavily on AU's scientific review process, though it can determine which conditions to include, thus ensuring that any decisions adopted from AU fits the NZ context and veteran population. A Clinical Advisor from NZ is invited to AU's deliberations and provides advice on the scientific-medical evidence for the Statements of Principles as to whether the evidence is relevant to the NZ Defense Force. The Clinical Advisor is also a member of the NZ Veterans' Health Advisory Panel, which advises whether the Statements of Principles should be adopted by NZ. For example, the NZ Defense Force has many Maori and Pacific peoples as members and it is expected that any differences that may impact on medical conditions be taken into consideration. The presence of experts from numerous fields on the panel (e.g., epidemiology, psychology, socioeconomics, and medical practice) also promotes a comprehensive evidentiary review prior to decision making.

## Discussion

Across the four countries summarized, presumptive condition lists vary by the conditions included, the military requirements for eligibility, and the evidentiary review processes used to develop these lists and exposure criteria. This is not unexpected, given national differences in domestic environments and international deployments that can impact risk arising from hazardous exposures (21).

### The Role of Scientific Review

A notable consistency across countries is the presence of scientific review in the development of presumptive condition lists. A comprehensive review of available scientific evidence is an essential step to evidence-based decision making and, when communicated effectively, can positively impact public trust (22). Scientific advisory panels or other external scientific advice mechanisms that assess the current state of evidence on a given condition and its relationship to military service play an important role in processes to develop or update presumptive lists, as well as other decisions concerning causation and military-relatedness. Internal/external membership representing a range of fields of expertise may independently conduct an evidentiary review (e.g., AU), assess the applicability of decisions in other jurisdictions to their own contexts (e.g., NZ) or commission external agencies to conduct reviews on their behalf (e.g., US). Criteria guiding the composition of such scientific committees typically include medical and scientific expertise in various health fields. For example, Australian legislation detailing the composition of the Australian Repatriation Medical Authority states “one of the (5) members must be a person having at least 5 years of experience in the field of epidemiology” with the committee's Chairperson being “a registered medical practitioner, or medical scientist, with at least 10 years of experience” (23). Similar requirements have been noted in civilian worker compensation contexts, where the majority of scientific/advisory committee members require “medical and scientific expertise” in backgrounds including occupational medicine, environmental health, occupational hygiene, epidemiology, and toxicology (24).

### Scientific Principles

Scientific principles to consider during the development and expansion of presumptive condition lists have been discussed in detail elsewhere (25). One important consideration is the existence of strong scientific evidence to establish a causal connection between one or more relevant exposures during military service and the health condition. Findings from national and international agencies and organizations tasked with assessing links between chemicals, forms of radiation, or other factors with long-term disease (such as the US National Academy of Sciences and the International Agency for Research on Cancer) are often referred to. Systematic reviews, or multiple high-quality studies showing a causal relationship between the disease and the military-related exposure, may also be used to guide decision making.

Clear diagnostic criteria are also important, to minimize doubt that the claimant has the condition in question (straightforward for some conditions, such as cancers, but less so for others, such as syndromes). It is also preferable for a presumptive schedule to be structured around conditions of interest with qualifications as to which exposures should be considered as causal, rather than around a particular exposure. For example, exposure to chromium has been linked to a number of chronic diseases and respiratory and skin disorders that may also be caused by agents other than chromium. “Lung cancer caused by exposure to chrome or its toxic compounds” provides clearer guidance as compared to “Diseases arising due to exposure to chrome or its toxic compounds”. Finally, it is relevant to consider the contribution of military factors to the burden of the condition in question, which can be examined by, for example, comparing age-adjusted rates in veterans to their civilian counterparts (26).

### Knowledge Transfer Across Veteran Compensation Systems

As stated previously, knowledge sharing across veterans' administrations with similar priorities may support program efficiencies *via* leveraging of external experience and resources. Therefore, presumptive condition lists and procedures developed by other jurisdictions may provide a useful starting point for other countries with similar priorities. However, it is important to examine the applicability and validity of other lists to the military population of interest, with consideration of cross-country differences in environmental and operational contexts (e.g., recruitment, training and operational policies, domestic environments, and deployments). It has been observed, for example, that even within a specific conflict the risk of hazardous exposures can differ according to the extent of a country's contribution of navy, army, and air force personnel (21). Domestic environments also vary; for example, the high prevalence of skin condition benefits administered to Australian Veterans may not be relevant to northern countries, where solar UV exposure is less of a domestic concern for military personnel.

### Knowledge Transfer Across Veteran and Civilian Systems

In addition to exchange across veterans' administrations, it is relevant to examine the evidentiary basis of deemed disease lists in civilian worker compensation systems, since many workplace hazards are common to both civilian and military environments. There has been a historic separation of compensation for civilian and veteran populations in need of assistance, often through parallel systems governed by different principles and rules (27). However, there are also many similarities, since both systems must review and weigh various types of evidence to determine the relatedness of a claimed condition to military service or civilian work. Instances of cross-system benefits have also been demonstrated, such as the Canadian example of federal subsidies to rehabilitate spinal-cord-injured veterans from the Second World War period leading to expanded disability policies and programs for civilians (28).

Scientific concepts that underpin deemed disease lists in civilian systems may also be informative to veteran systems. In Australia for example, Section 7 of the Safety, Rehabilitation and Compensation (Defense-related claims) Act 1988 (29) states provisions to accept certain condition claims on the basis of defined occupational exposures. These conditions are specified in a legislative act primarily related to a civilian context (30). The updated deemed diseases list as of 2017 is based on Safe Work Australia's Deemed Diseases in Australia (31), a recommended list of deemed diseases based on a detailed external review of published scientific information and the application of three required criteria: strong causal link between the disease and occupational exposure; clear diagnostic criteria for the disorder; and the diseases needing to comprise a considerable proportion of cases in the overall population (or identifiable subset of).

In the UK, civilian occupational conditions are compensated under the Industrial Injuries Scheme. Benefits are payable to workers with a prescribed condition that is listed in the Prescribed Diseases Regulations (32). The Prescribed Diseases List is updated on the advice of the Industrial Injuries Advisory Council, an independent scientific advisory body created in 1948 to advise the government on matters related to the administration of the Industrial Injuries Scheme [e.g., (33)].

### Future Directions

This article provides insight into presumptive condition lists (or their equivalent) in veteran benefits systems of four primarily English-speaking countries. Extending this scan to systems in other countries would provide more information, and perhaps greater variability, in presumptive condition lists and processes used to develop them. A number of related issues also merit further investigation, including procedures undertaken to review scientific evidence and make determinations of causality, and as well as broader legal, political, and administrative considerations that impact decision making.

Prior studies of exposure and health surveillance and record keeping in the military context, particularly in deployed combat settings, illustrate both opportunities and limits of “big data” systems to inform research, prevention, and compensation of service-related injury and disease (34, 35). Support for ongoing research, including administrative linkages, is needed to strengthen this evidence base (2). While the establishment of clear criteria for exposure dose/duration is a challenge for many conditions, it is interesting to note that AU's approach to streamlining conditions for veteran compensation has undergone regular enhancements as additional data collection and research strategies have been undertaken. For example, significant work was carried out to collect and analyze physical training program data from each of the entry schools to the Australian Defense Force to develop streamlined condition processes (36).

Research focused on specific subgroups (e.g., females and other minorities) is also important to understand potential differences in exposures and susceptibilities, and implications for compensation rules and decisions. Such information can be directly applied in decision-making, for instance NZ's review and adaptation of AU's streamlined conditions considers unique characteristics of Maori and other Pacific populations and potential impacts on medical conditions in veterans.

## Conclusions

Compensation policies carry broad impacts on claimants and government agencies responsible for veteran wellbeing. The establishment of presumptive condition lists is complex, particularly when attempting to assess condition causation and military-relatedness. This environmental scan of four countries identified a range of health conditions covered, military requirements for eligibility, and scientific review processes used to develop presumptive condition lists. Opportunities to leverage evidence and experience across veteran, as well as civilian, systems should be considered. Ongoing research to understand links between exposures and health outcomes in military populations is also recommended.

## Author Contributions

AH designed the study and drafted the manuscript. MD and AH performed data collection. All authors interpreted the study results, contributed to manuscript revisions, approved the final version for submission, and agree to be accountable for all aspects of the work.

## Funding

This study was conducted with internal funding at Veterans Affairs Canada.

## Conflict of Interest

The authors declare that the research was conducted in the absence of any commercial or financial relationships that could be construed as a potential conflict of interest.

## Publisher's Note

All claims expressed in this article are solely those of the authors and do not necessarily represent those of their affiliated organizations, or those of the publisher, the editors and the reviewers. Any product that may be evaluated in this article, or claim that may be made by its manufacturer, is not guaranteed or endorsed by the publisher.
